# The first autochthonous case of feline ocular thelaziosis in Austria

**DOI:** 10.1007/s00436-019-06275-0

**Published:** 2019-03-02

**Authors:** Adnan Hodžić, Albert Payer, Georg Gerhard Duscher

**Affiliations:** 1 Institute of Parasitology, Department of Pathobiology, University of Veterinary Medicine Vienna, Veterinaerplatz 1, 1210 Vienna, Austria; 2 Tierklinik Deutschlandsberg, Erlenweg 6, 8530 Deutschlandsberg, Austria

**Keywords:** Austria, Cat, *cox*1, Haplotype 1, *Thelazia callipaeda*

## Abstract

Over the last 30 years, *Thelazia callipaeda* (Spirurida: Thelaziidae) has increasingly been reported as an agent of ocular infections in animals and humans throughout Europe. Following the cases of canine ocular thelaziosis recently recorded in Austria for the first time, in the present paper, we describe the first case of *T. callipaeda* infection in an Austrian cat with no history of traveling abroad. This finding further supports the occurrence of the parasite’s autochthonous transmission cycle in the country. The cat showed serous ocular discharge, conjunctival hyperemia, and mild conjunctival edema in the right eye. Mechanical removal of the parasite from the cat’s eye, in combination with milbemycin oxime/praziquantel oral treatment and topical use of tobramycin/dexamethasone eye drops led to complete resolution of the clinical signs within 2 weeks. Results presented in the current study are of great importance for the local veterinarians who seemed largely unaware of this zoonotic parasite. Therefore, increased awareness of medical and veterinary communities is imperative for preventing further infections in both animals and humans.

## Introduction

**Fig. 1:**
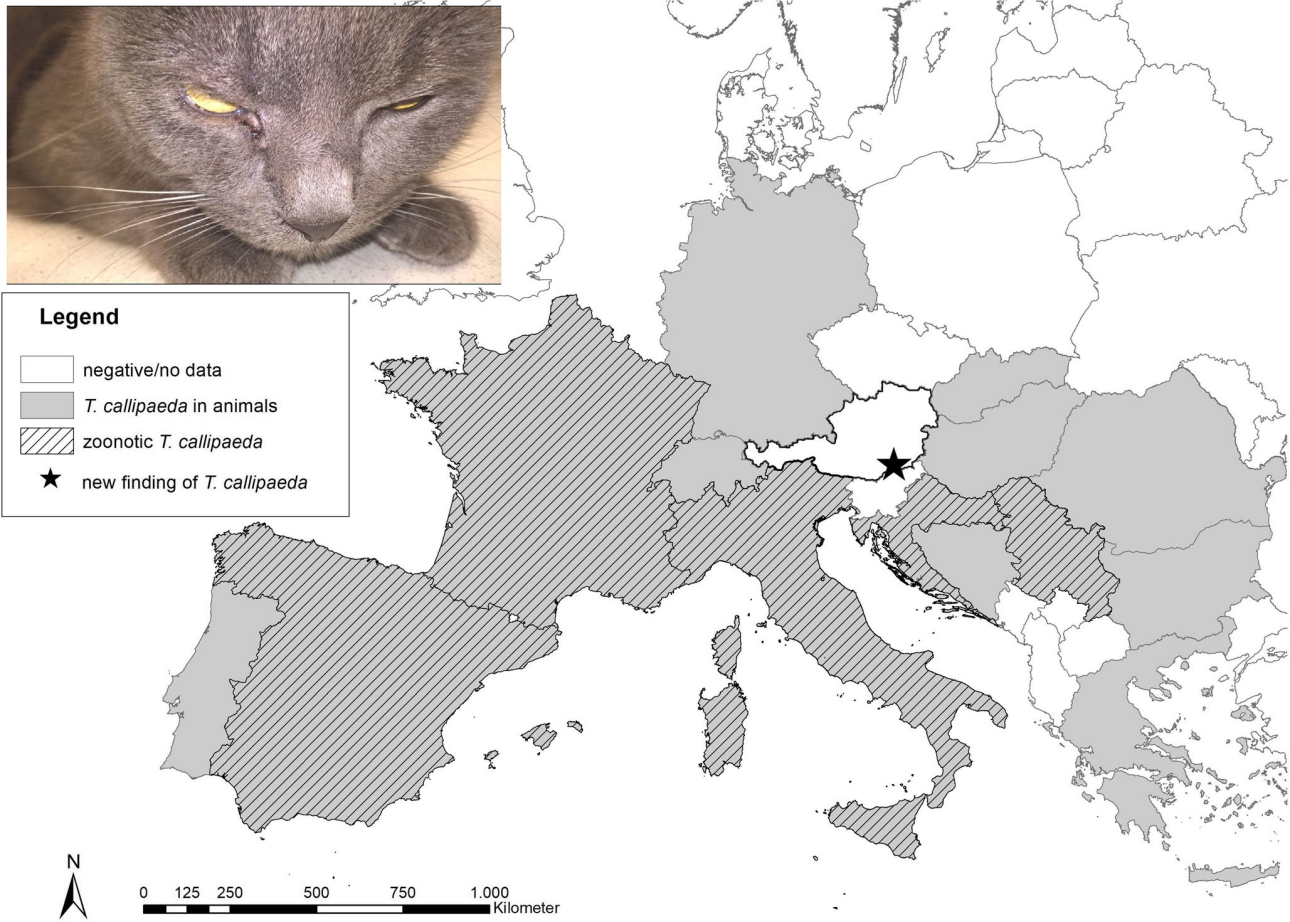
A map showing the distribution of known cases of *Thelazia callipaeda* infections in Europe. Black star indicates the location where the infected cat in Austria was found

*Thelazia callipaeda* (Spirurida, Thelaziidae) is a vector-borne nematode of veterinary and medical concerns inhabiting the conjunctival sac and associated ocular tissues of domestic and wild carnivores, lagomorphs, and humans (Otranto et al. 2009, 2015). This zoonotic parasite is capable to induce a variety of clinical signs in the infected hosts, ranging from asymptomatic carriage to mild and severe ocular pathology that includes, e.g., epiphora, blepharitis, conjunctivitis, and keratitis (Otranto and Traversa 2005). In Europe, *T. callipaeda* is transmitted by male *Phortica variegata* (Drosophilidae, Steganinae) drosophilids, which deposit infective third-stage larvae while feeding on ocular secretions of the hosts (Otranto et al. 2006a). Because the nematode originally occurred in the Far Eastern countries, it has often been referred to as “oriental eye worm” (Máca and Otranto 2014). However, since the first European case of canine ocular thelaziosis was described in Italy in 1989 (Rossi and Bertaglia 1989), *T. callipaeda* has increasingly been reported in animals from France (Dorchies et al. 2007), Switzerland (Malacrida et al. 2008), Germany (Magnis et al. 2010), Spain (Miró et al. 2011), Portugal (Vieira et al. 2012), Bosnia and Herzegovina (Hodžić et al. 2014), Croatia (Hodžić et al. 2014), Serbia (Gajić et al. 2014), Romania (Mihalca et al. 2015), Bulgaria (Colella et al. 2016), Hungary (Colella et al. 2016), Slovakia (Čabanová et al. 2017), and Greece (Diakou et al. 2015; Papadopoulos et al. 2018) (Fig. 1). The zoonotic potential of this nematode has been confirmed by several human cases recorded in endemic regions of Europe (Otranto and Dutto 2008; Fuentes et al. 2011; Tasić-Otašević et al. 2016; Paradžik et al. 2016) (Fig. 1).

Canine infections caused by *T. callipaeda* have recently been described in Austria for the first time, with only five cases reported by the local veterinarians over the last 5 years (Hinney et al. 2016). Three of the cases were presumably autochthonous since the dogs likely had never left the country. In this paper, we describe the first case of feline ocular thelaziosis due to *T. callipaeda* in Austria.

## Case report

In November 2018, a six-year-old neutered male European shorthair cat, suffering from chronic conjunctivitis of the right eye, was referred to its local veterinary clinic in Deutschlandsberg, Austria (coordinates, 46° 48′ 58″ N, 15° 12′ 54″ E) (Fig. 1). According to anamnestic data, first signs of ocular disease appeared 4–5 weeks before the cat was brought to the clinic. Ophthalmological examination revealed unilateral serous ocular discharge, conjunctival hyperemia, and mild conjunctival edema. Additionally, a thread-like motile worm was noticed under the nictitating membrane of the right eye. No other ocular abnormalities were detected. The parasite was retrieved using a forceps, placed in a tube with saline solution and sent to the Institute of Parasitology, University of Veterinary Medicine Vienna for morphological and molecular identification.

After removing the parasite, the cat was orally treated with milbemycin oxime 2 mg/kg and praziquantel 5 mg/kg (Milbemax®, Novartis Animal Health, France). In addition, tobramycin 3 mg/ml and dexamethasone 1 mg/ml eye drops (Tobradex®, Alcon Ophthalmika, Austria) were administrated twice a day in a 7-day treatment course. At a follow-up 2 weeks after the treatment, complete resolution of clinical signs was observed and no parasites were detected.

The nematode was identified as a female of *T. callipaeda* based on the specific morphological features (e.g., striated cuticula, hexagonal oral opening, vulva located anteriorly to the esophageal-intestinal junction, uterus filled with larvated eggs) (Otranto et al. 2004a). Species identity was further confirmed by PCR amplification and sequencing of the cytochrome *c* oxidase subunit 1 (*cox*1) gene (Otranto et al. 2005). Nucleotide sequences derived from the adult nematode displayed 100% identity to the *cox*1 sequences of *T. callipaeda* haplotype-1 (GenBank® accession no. AM042549), which is the only haplotype circulating among animals and humans in Europe.

## Discussion

Following the recent reports of canine thelaziosis in Austria (Hinney et al. 2016), the current study describes the first case of *T. callipaeda* infection in a cat that never traveled abroad, further supporting the existence of autochthonous transmission cycle in the country.

However, the way(s) how this parasite was initially introduced to Austria remains unknown. One of the possible explanations is that *T. callipaeda* could have arrived via pet traveling, illegal pet trade or import/export of stray dogs, usually coming from endemic regions of Eastern Europe. These have often been indicated as efficient routes for transmission and spread of zoonotic parasites from endemic to non-endemic areas (Graham-Brown et al. 2017). For instance, three cases of imported *T. callipaeda* infections had recently been described in dogs from the United Kingdom that traveled to the countries in Europe where the parasite is highly prevalent (Graham-Brown et al. 2017).

Furthermore, the geographical expansion of *T. callipaeda* to non-endemic areas has also been linked to the migration of infected wild carnivores, in particular foxes, which seem to play an important role as reservoirs of *T. callipaeda* in Europe (Otranto et al. 2009; Hodžić et al. 2014; Mihalca et al. 2016). This is also a plausible scenario which could explain the introduction of the eye worm to Austria. Therefore, future studies should be focused on wild animals in order to assess the role they play in the eco-epidemiology of this zoonotic parasite.

*Thelazia callipaeda* is a vector-borne nematode and thus its distribution is expected to overlap with dispersion range of the fruit fly *P. variegata*, the only known vector of this nematode in Europe (Máca and Otranto 2014). However, the introduction of the parasite from abroad by the vector seems to be less likely because fruit flies are not able to travel over long distances (Ruytoor et al. 2010). To the best of our knowledge, the occurrence of *P. variegata* has not yet been confirmed in Austria. Meanwhile, the ecological niche model, used to predict the distribution of this fruit fly, showed that large areas of Austria, including the place where the infected cat was found, is environmentally suitable for the development of *P. variegata* and consequently for establishing endemicity (Otranto et al. 2006b; Palfreyman et al. 2018). The infected cat was living indoor, with regular outdoor access, in a rural area characterized by many vineyards and wildlife species richness, which represents the typical environment requested by the parasite to complete its life cycle. Moreover, the cat from the presented case was referred to the veterinary clinic in November, but it displayed signs of ocular discomfort a month before that. Considering the parasite requires several weeks to reach the adult stage (Otranto et al. 2004b, 2005), which is mostly responsible for causing clinical signs of the infection, it could be presumed that the animal acquired the parasite in summer (July–August) when the vector activity is at the highest level (Otranto et al. 2006a, b). In any case, it is reasonable to expect more cases of ocular thelaziosis in the following years and its further expansion to new areas in Austria, as recently demonstrated in a study conducted in Spain (Marino et al. 2018).

Treatment of *T. callipaeda* infection includes mechanical removal of the worms and administration of macrocyclic lactones. Oral milbemycin oxime/praziquantel and milbemycin oxime/afoxolaner combinations (Motta et al. 2012; Lebon et al. 2019), as well as moxidectin/imidacloprid spot-on formulation (Otranto et al. 2016, 2019) showed high therapeutic efficacy in naturally infected dogs and cats. Milbemycin oxime/praziquantel treatment should be given twice in an interval of 7 days (Motta et al. 2012), whereas a single treatment of moxidectin/imidacloprid and milbemycin oxime/afoxolaner is found to be sufficient for complete parasite elimination (Otranto et al. 2016, 2019; Lebon et al. 2019). Moreover, the monthly administration of the abovementioned anthelmintic products can be used as a prophylactic treatment (Motta et al. 2012; Otranto et al. 2016, 2019; Lebon et al. 2019). In this study, mechanical removal of the parasite followed by oral administration of milbemycin oxime/praziquantel led to complete resolution of the associated clinical signs in 2 weeks.

Results presented in the current study are of great importance for the local veterinarians who seemed largely unaware of this zoonotic parasite. Therefore, increased awareness of medical and veterinary communities should help to prevent further infections in animals and humans.
